# Reconsidering performance management to support innovative changes in health care services

**DOI:** 10.1108/JHOM-12-2022-0379

**Published:** 2024-03-22

**Authors:** Anell Anders

**Affiliations:** Department of Business Administration, Lund University School of Economics and Management, Lund, Sweden

**Keywords:** Health care delivery, Innovative change, Enabling control, Performance management, Feedback, Professionalism, Nonfinancial incentives, Motivation

## Abstract

**Purpose:**

A large number of studies indicate that coercive forms of organizational control and performance management in health care services often backfire and initiate dysfunctional consequences. The purpose of this article is to discuss new approaches to performance management in health care services when the purpose is to support innovative changes in the delivery of services.

**Design/methodology/approach:**

The article represents cross-boundary work as the theoretical and empirical material used to discuss and reconsider performance management comes from several relevant research disciplines, including systematic reviews of audit and feedback interventions in health care and extant theories of human motivation and organizational control.

**Findings:**

An enabling approach to performance management in health care services can potentially contribute to innovative changes. Key design elements to operationalize such an approach are a formative and learning-oriented use of performance measures, an appeal to self- and social-approval mechanisms when providing feedback and support for local goals and action plans that fit specific conditions and challenges.

**Originality/value:**

The article suggests how to operationalize an enabling approach to performance management in health care services. The framework is consistent with new governance and managerial approaches emerging in public sector organizations more generally, supporting a higher degree of professional autonomy and the use of nonfinancial incentives.

## 1. Background

Performance measurement is the regular collection and feedback of data concerning resources, activities, results and outcomes for an individual, team or organization (Neely *et al*., 2005). Such collection and feedback of data can support different purposes and forms of control (Franco-Santos *et al*., 2012) and serve diverse functions across different stakeholders in the public sector (Johnsen, 2005). Performance measurement and accountability towards targets are highly visible components of New Public Management (NPM) reforms initiated in the late 1970s (Hood, 1991) and since then widely introduced in Anglo-Saxon and European public and health sectors (Diefenbach, 2009; Arnaboldi *et al*., 2015; Siverbo *et al*., 2019).

While the early NPM reforms in health sectors focused on efficiency in a narrow sense, e.g. cost per discharge from hospitals, reforms in the new millennium have a greater focus on quality and value-for-money (Cutler, 2002; Smith *et al*., 2012). A greater focus on quality measures has contributed to changing margins of organizational control (Miller, 1998; Pflueger, 2016, 2020) and has been used to extend the reform agenda through quality-based competition and pay-for-performance schemes (P4P) (Porter and Teisberg, 2006; Porter, 2009). This use of quality measures linked to financial incentives and for external accountability have often ignored that monitoring of quality has existed for a long time in health care services, the purpose being to support internal quality improvement work (Braspenning *et al*., 2013). “Internal” here refers to how professional´s use the data; to monitor the outcome of interventions, to learn and to identify best practice. With a growing interest in quality measures by payers, politicians and general managers, many different voices exist on how measures should be used (Østergren, 2006). Data in medical quality registers are no longer solely about learning and of internal concern for professionals (Funck, 2015).

From the perspective of health professionals, performance measurement to support external accountability constitutes a paradigm shift in the use of (their) data. Frequently, health care professionals have described monitoring of quality measures for external accountability as an administrative burden with limited benefits, arguing that such use limits their professional autonomy and motivation (McDonald and Roland, 2009; Young *et al*., 2017) and contributes to burnout (Bodenheimer and Sinsky, 2014). Reviews of empirical studies suggest that quality-based competition have had a limited effect on the quality of care (Fotaki, 2020; Van Ginneken *et al*., 2020). Likewise, P4P schemes usually have limited effects on process measures and no effect on outcome measures (Van Herck *et al*., 2010; Scott *et al*., 2011; Eijkenaar *et al*., 2013; Ogundeji *et al*., 2016; Ellegård *et al*., 2018). Additional and important criticism from a performance management perspective is that quality measures are assessed in isolation and seen as providing final and summative answers when used for external accountability and coercive forms of control (Arnaboldi *et al*., 2015). In practice, quality measures are often incomplete (Berwick, 2009; Young *et al*., 2017) which means that they need to be evaluated in combination and together with contextual information using professional judgment. Value conflicts when measuring performance in the public sector have been reported to create “creative destruction” (Johnsen, 2005) but may also crowd out public service motivation (Frey *et al*., 2013; Ritz *et al*., 2016) and destabilize the identity of professionals (Skaerbeck and Thorbjörnsen, 2007). Indeed, some P4P studies report unintended effects, e.g. that providers manipulate data and that changes in behavior are not to the benefit of patients (McDonald and Roland, 2009; Eijkenaar *et al*., 2013; Bevan *et al*., 2019).

Against the background of reported problems and limited benefits, new perspectives when it comes to performance management in health care services are called for. These new perspectives need to consider contemporary challenges. Challenges include a growing burden of disease, staff shortages and rapid technological development including digital and e-health solutions (see, e.g. Topol, 2019; Britnell, 2019; OECD, 2019; National Academies of Sciences Engineering and Medicine, 2021). In combination, these challenges both require and create opportunities for innovative changes in the delivery of services (National Academies of Sciences Engineering and Medicine, 2021). Against this background, the main purpose of this article is to explore and discuss new approaches to performance management in health care services that are able to support innovative changes in the delivery of services.

Methodologically, the article represent cross-boundary work as the theories and empirical findings used to discuss and explore new approaches to performance management comes from several separate research disciplines, including studies of organizational control, systematic reviews of clinical audit and feedback interventions and cognitive theories of motivation. The article attempts to bring together strands of research that have not been talking to each other so far, the aim being to shed new perspectives on an old and contested issue. The broad approach also comes with methodological challenges regarding the selection of theories and empirical reviews in different sections as well as its synthesis. The purpose of Section 2 is to describe why an enabling rather than a coercive approach to performance management is needed when supporting innovative change in the delivery of services. The following Sections 3 through 6 focus on how to operationalize the approach suggested in Section 2. In Section 3, main lessons from empirical studies of clinical audit and feedback interventions are summarized. The search strategy used focused on identification of systematic reviews, starting with reports in the Cochrane Database. Additional articles, in particular addressing behavior change techniques used in interventions, were identified from a forward citation search. Section 4 explores how determinants of motivation and nonfinancial incentives relate to performance management in the context of health care services more generally. The starting point of this section is extant and general theories of motivation in a work context and how these theories apply to the context of health care services. This theoretical review complement identified lessons from empirical studies of audit and feedback interventions, often described as limited when it comes to use of behavioral science frameworks and theory (Crawshaw *et al*., 2023; Davidoff *et al*., 2015; Colquhoun *et al*., 2013). In Section 5, design elements of an enabling approach to performance management is discussed with reference to a synthesis of the material in sections three and four. Section 6, finally, discuss key challenges when implementing the new approach.

## 2. How can performance management support innovative changes?

A specific challenge when supporting innovative change is the role and involvement of “top-management” vs the operational core (Davila *et al*., 2009). As innovative changes in the delivery of health care services often involve complexity, this article will assume that some form of bottom-up approach is required. This assumption is not without reasons. Complex changes contain several interacting components (Hawe, 2015) creating barriers to replication and scalability (Horton *et al*., 2018). Top-management can ask for more teamwork, task shifting and collaborations but complex changes works best if tailored to local conditions rather than being completely standardized (Craig *et al*., 2008). Moreover, innovative changes in health care are usually dependent on commitment from the operational core, which further favors a bottom-up approach. For these reasons, the type of organizational control exercised need to have an enabling approach (Adler and Borys, 1997; Ahrens and Chapman, 2004). This contrasts common practice of performance management in public health care services, tending to apply coercive forms of control (Abernethy *et al*., 2007; Bevan and Hood, 2006; Smith *et al*., 2012).

To clarify how an enabling approach to performance management can support innovative changes, the two “modes” of innovation discussed by Jensen *et al*. (2007) are helpful. The linear Science, Technology and Innovation (STI) mode is the predominant perspective in the professional core of health care services. Innovations are developed by someone else, e.g. by university hospitals or pharmaceutical companies. Health technology assessment (HTA) agencies develop evidence-based guidelines. The role of performance management is to support implementation of these standards; to provide regular information about gaps between clinical practice and best available evidence. The type of control used will consequently be rather coercive. A main difference from organizational control is that professionals themselves, or at least the professional elite, determines goals and targets, rather than managers belonging to the administrative logic. In contrast, the Doing, Using and Interaction (DUI) mode is oriented towards learning and improvement in a local service delivery context that also emphasize practical experiences. If performance management is to support a DUI mode of innovation, professionals and operational managers should be able to formulate goals and action plans based on a formative use of measures (Jensen *et al*., 2007). Measures need to be combined with qualitative and experience-based information and assessment of local conditions and priorities, requiring dialog and involvement by professionals (Davies, 2005). This is in sharp contrast to a summative approach that consider feedback messages as final answers. An enabling approach suggests that performance measurement and feedback messages should be viewed as a “learning machine” rather than an “answering machine” (Abernethy and Brownell, 1999; Abernethy *et al.*, 2007).

The STI and DUI approach are not necessarily antagonistic in practice (Isaksen and Nilsson, 2013). The DUI approach does not exclude that providers at the same time are recipients of STI innovations. Demands for compliance can be strict in some sense, e.g. treatment guidelines for a particular patient group, whereas local goals and action plans are favored to support development of innovative changes in the delivery of services. Several STI innovations need to be adapted to the local context, i.e. they require specific development using a DUI approach. The preferred combination between STI and DUI modes of innovation is likely to vary depending on the setting. A DUI mode and enabling approach to performance management will be more important if evidence related to delivery of services does not exist or if conditions vary, making it more difficult to rely on general standards and more important to initiate learning and development locally. Compliance towards incomplete measures may engage providers in a “box-ticking” behavior not likely to benefit patients (Maisey *et al*., 2008; Campbell *et al*., 2008) and is usually associated with higher levels of randomness making a summative use of measures more difficult (Lilford *et al*., 2004; Petersen *et al*., 2006).

## 3. Lessons from evaluations of clinical audit and feedback interventions

Feedback to users and decision makers is an important component of any performance management system. In the practice of clinical audit and feedback interventions, the main recipients of feedback messages are usually individual physicians or small provider teams (Crawshaw *et al*., 2023). Feedback usually focus process measures and comparisons with peers and targets; reports are frequently combined with face-to-face meetings with possibilities to discuss data (Colquhoun *et al*., 2017). As nonfinancial clinical audit and feedback interventions have existed for long and is generally accepted by professionals, evidence related to “what works and not” should be of interest from a general performance management perspective.

The latest available Cochrane Systematic Review of 140 randomized and controlled trials (Ivers *et al*., 2012) point to a positive although varying impact on professional´s behavior. This is an important lesson from a performance management perspective; change is possible without using financial incentives. Perhaps even more important and in sharp contrast to studies of financial incentives, reports of dysfunctional consequences are virtually non-existent.

More specifically, findings from the latest Cochrane review continues to be frequently cited and used in several guidelines (Ivers *et al*., 2022). Previous policy recommendations based on findings from the review (Ivers *et al*., 2014; Brehaut *et al*., 2016) suggest that clinical audit and feedback should:

1. use validated and up-to-date data concerning the individual or team in focus;
2. be provided regularly in multimodal forms (text-based feedback, visualization aids and face-to-face meetings);
3. be provided by a trusted and legitimate source (supervisor or colleague);
4. include comparison with other relevant practices and targets and
5. provide linkages to action plans.

Even when clinical audit and feedback interventions follow this advice a positive impact is far from certain, however. The Cochrane review summarized that impact depends on contextual factors such as recipient´s capabilities and motivation as well as existing cultural, organizational and regulatory opportunities for change. The review also revealed a more visible impact among providers with poor performance and if the required change was simple, i.e. if physicians and recipients of feedback could implement changes individually.

An ongoing update of the Cochrane review, to include a total of 287 randomized trials up to June 2020, aims to further explore factors that explain the effectiveness of audit and feedback (Ivers *et al*., 2022). A review by Colquhoun *et al*. (2017) indeed identified no less than 17 modifiable design elements of clinical audit and feedback interventions. A descriptive article from the Cochrane update found that the most used behavior change techniques in the 287 trials was providing feedback on behavior, sharing guidelines on appropriate behavior, comparison of behavior with peers, endorsement of feedback and guidelines from a professional body and educational activities (Crawshaw *et al*., 2023). Additional analysis of the relationship between these behavioral techniques and effects based on findings from the 287 trials have so far not been published (December 2023). Studies identified from a forward citation search since the latest available Cochrane review makes it clear that both design and context matters, however. Laboratory experiments and field studies indicate that the impact of clinical audit and feedback depend on whether recipients trust the data, agree with targets and benchmarks and consider the topic important (Gude *et al*., 2017, 2018). Although recipients have an intention to change, i.e. they are informed and have the capability and motivation to change, actual change also require opportunity (Landis-Lewis *et al*., 2015) and depends on if recipients deem improvement feasible (Gude *et al*., 2017, 2018).

The Cochrane reviews as well as the additional studies referred to clearly views clinical audit and feedback interventions from an “implementation” or “diffusion of innovation” perspective. The purpose is to close the gap between medical evidence and clinical practice (Colquhoun *et al*., 2013). It is assumed that exogenous and evidence-based targets exist and that care providers should comply with standards, at least for most of their patients. With reference to Section 2 in this article, this means that clinical audit and feedback usually follow the STI mode of innovation, trying to coerce professional behavior towards evidence-based standards. Moreover, the focus of interventions is usually simple changes that can be implemented by individual professionals, such as prescribing, testing and/or treatment decisions (Crawshaw *et al*., 2023). Complex changes are more demanding, requiring that recipients perceive that collective improvement work is feasible. Parallel interventions to influence behavior and removal of organizational barriers can then make a huge difference. Recipients may ignore feedback messages suggesting complex changes involving task shifting from doctors to nurses, collaboration with others or adaption of e-health solutions if payment systems do not support such changes. Even if feedback interventions themselves are nonfinancial, their impact may consequently depend on financial incentives. These are all important limitations. Performance management that aims to enable more complex and innovative changes in the delivery of health care services can learn from but should avoid copying what may work in a clinical audit and feedback context.

## 4. Performance management and determinants of motivation

While the assumption in quality-based competition and P4P schemes is that providers need financial incentives to change their behavior, clinical audit and feedback interventions are rather silent when it comes to *why* recipients would be motivated to initiate change following feedback messages alone. The explicit use of behavioral science frameworks and theory in empirical research of audit and feedback interventions have been described as limited (Crawshaw *et al*., 2023; Colquhoun *et al*., 2013). Studies often assume that recipients of feedback will develop an intent to change as they become aware of deviations and gaps, i.e. motivation to change comes from discrepancies as such (Kluger and DeNisi, 1996; Locke and Latham, 2002; Harmon-Jones and Mills, 2019). The same conclusion has been made for improvement research in general. If theories are used, they can best be described as frameworks or “middle-range theories”, limited to the areas of application and used to unpack the relationship between interventions and effects, rather than to explain the motivation to change (Davidoff *et al*., 2015). In this section, extant and “grand” theories of motivation and how these apply to innovative changes in health care services will be discussed. The purpose is to gain a deeper understanding of why individuals in a work context may be motivated or not to change, thereby complementing identified lessons from empirical studies of audit and feedback interventions presented in Section 3.

Management studies addressing a purposeful design of performance management systems often depart from assumptions about individual´s motivation based on agency theory (Abernethy *et al*., 2007). These assumptions imply that employees need to be regulated and extrinsically incentivized to curb opportunistic behavior (Gneezy *et al*., 2011). Although such assumptions can be reasonable when addressing economic transactions, they are less valid in contexts characterized by social interaction (Fehr and Falk, 2002), for qualitative type of tasks (Cerasoli *et al*., 2014) and when facing uncertainty and goal ambiguity (Abernethy *et al*., 2007). A large number of empirical studies confirm that there is a “dark side” of financial incentives attached to coercive forms of control, in particular when used in public services (Frey and Jegen, 2001; Frey *et al*., 2013) and for complex tasks (Cerasoli *et al*., 2014). Abernethy *et al*. (2007) describes how “the particular features of the health care sector make it an ideal laboratory in which to study how the implementation of accounting systems can result in unintended consequences” (p. 810).

While agency theory views the world through a lens of economic transactions between principals and agents, an enabling approach to organizational control and performance management need to view the world through a lens of social interactions between humans. From the human side, work-related motivation has many determinants and a complex relationship with incentives exists that may backfire (Fehr and Falk, 2002; Gneezy *et al*., 2011). Besides extrinsic incentives in the form of separable rewards and sanctions, intrinsic motivation from the task itself as well as self- and social-approval mechanisms need to be fully recognized. For qualitative type of tasks, intrinsic motivation from the joy of performing the task is important (Ryan and Deci, 2000; Cerasoli *et al*., 2014). Intrinsic motivation can be facilitated through task design (i.e. making the job more interesting) a continued development of competence and support of autonomy. Employees perception of the locus of control (i.e. if behavior is perceived to be self-controlled or not) is fundamental for intrinsic motivation (Ryan and Deci, 2000) and directly influenced by the design of extrinsic incentives (Cerasoli *et al*., 2014). Use of direct financial incentives, e.g. P4P schemes focusing process measures, influence the perceived locus of control negatively. To avoid crowding out of intrinsic motivation and approval mechanisms, incentives should not be contingent on certain task behavior (Frey and Jegen, 2001).

Intrinsic motivation is frequently recognized in studies of public services, but often used in a broader sense that includes what public servants think about themselves and their contribution to society (see, e.g. Ritz *et al*., 2016). In practice, public servants may think they are doing something important for society even if their task is simple. Public servants may also be motivated by a complex task, even if the task is not valued by the society. From a cognitive behavioral perspective, self- and social-approval mechanisms should be viewed as separate determinants of motivation. Individuals in general have a deep imprinted desire to seek approval and avoid disapproval in relations to others, not least from individuals and groups that they identify with and look up to (Fehr and Falk, 2002). Individuals also care about their identity and what they think about themselves. A continual process of social- and self-approval influence individual´s feelings of pride and shame (Ellingsen and Johanesson, 2007, 2008). These mechanisms – to seek pride and avoid shame–explain why feedback messages itself can influence behavior, in particular when feedback focus measures that individuals think are important, if received from a legitimate and trusted source and among providers with poor performance; the latter providers are likely to experience more dissonance (and shame) related to their professional identity. The same mechanisms also explain why individuals can act in unselfish ways, even when not observed by others. Individuals in general feel pride when performing altruistic actions and when being fair to others, although variation across individuals and depending on the context exist (Fehr and Falk, 2002). According to empirical studies, social determinants of motivation, altruistic preferences and fairness are particularly important in public services and professional service firms (Frey *et al*., 2013; Ritz *et al*., 2016).

Self- and social-approval mechanisms prevent individuals from being too opportunistic in relation to others. Indeed, if contracts are incomplete and difficult to monitor, principals may be better of trusting rather than controlling an agent (Falk and Kosfeld, 2006). The same mechanism becomes even more important in continuing social interactions, as it is then easier to initiate reciprocal actions towards unfair or selfish agents (Falk and Fischbacher, 2006). Employees expect fairness and respect from managers and co-workers when engaging in work-related interactions. Managers who act in selfish ways and are not treating others with respect may face reciprocal actions (Ellingsen and Johanesson, 2007). Such reciprocity includes employees resisting change and ignoring targets if managers are perceived as disrespectful, coercive and exploitative (Carpenter and Dolifka, 2017). In organizations that function well employees will identify and feel pride with their work and organization (Akerlof and Kranton, 2000, 2008). In organizations that function less well employees are more likely to create a distance towards the organization, develop an identity of their own and resist managerial interventions.

In summary, full consideration of intrinsic motivation and self- and social-approval mechanisms and tendencies by individuals to reciprocate if not experiencing recognition and respect, are particularly relevant for health care services with its strong professional orientation (Abernethy and Stoelwinder, 1995; Freidson, 2001). This does not imply that feedback of performance related to organizational objectives is irrelevant. Professionals confronted with views and interpretations from other stakeholders contribute by challenging status quo (Johnsen, 2005). In the absence of feedback, health professional´s tend to overestimate both their own as well as peer performance (Gude *et al*., 2018). From a cognitive perspective, however, it is always individuals’ perceptions of feedback messages and the following outcome in terms of individuals’ own goal-setting activities, that matter for performance (Locke and Latham, 1990, 2002, 2019; Latham, 2004). The active construction of discrepancies in a goal-setting process (Bandura and Locke, 2003; Wright, 2004) is likely to be even more important when supporting changes in healthcare services through a DUI mode of innovation. Performance management should then support development and commitment to local goals and action plans that fit specific conditions and challenges.

## 5. An alternative approach to performance management in health care services

In this section, design elements of an enabling approach to performance management are discussed with reference to the material presented in sections 2-4. Four interrelated design elements will be explored: (1) the choice of measures, (2) the use of measures and the development of local goals and action plans, (3) the source and modality of feedback messages and (4) the degree of transparency in feedback messages. Along with the exploration of these design elements, challenges for management are identified. The selection of design elements is based on the identified need for an enabling and bottom-up approach when supporting innovative changes in health care services. This approach means that local goals and action plans becomes more important; feedback messages should be viewed as an “learning machine” rather than an “answering machine” (Abernethy and Brownell, 1999; Abernethy *et al.*, 2007).

### 5.1 The choice of measures

To initiate change, professionals and their managers have to consider the performance measures used as valid, and they need to trust the data. Several studies recognize the active role of recipients and the importance of cognitive processes when providing feedback (Kluger and DeNisi, 1996; Locke and Latham, 2002; Harmon-Jones and Mills, 2019). Only deviations between actual performance and feedback messages that recipients accept and deem important will create a cognitive dissonance and a motive to act. To include and get acceptance of all relevant performance domains in health care services is challenging. From a professional perspective, clinical measures are most likely deemed as more important. From an organizational control perspective, measures related to organizational objectives and efficiency need to be considered. Providers with strong professional identities may ignore feedback or initiate defensive actions it they don´t recognize the measures used as relevant. Ignoring measures deemed as important only by “outsiders” (e.g. efficiency measures defined by the administrative body) may even strengthen the (professional) group identity and trigger reciprocal actions. Studies have shown that co-development of performance measurement systems together with employees increase understanding as well as organizational performance (Groen *et al*., 2012, 2017). The usual understanding of such co-development is that managers invite employees in the process of developing and choosing measures. How to organize a similar process of co-development and dialog in health care services, to support a balanced inclusion of relevant measures in feedback messages, is an important management challenge.

### 5.2 The use of measures and the development of local goals and action plans

As explained in Section 2, a DUI mode of innovation goes hand in hand with an enabling approach to performance management. Based on the design principles of enabling control (Ahrens and Chapman, 2004), this requires a more flexible use of standards. Managers together with professionals’ should be encouraged to formulate their own local goals and actions plans that fit specific conditions and challenges. A first requirement is that assigned goals are less controlling and not contingent on certain behaviors. Use of measures and feedback messages should be formative and learning oriented. With a focus on learning and change, accountability will be processual in nature (Virtanen *et al*., 2014), requiring a different leadership style compared to accountability towards predetermined standards.

A learning oriented use of measures is likely to contribute to higher levels of acceptance and commitment. Managers and professionals should be able to treat incomplete measures as means to increase understanding rather than ends when carrying out their work (Jordan and Messner, 2012). Alignment of feedback messages with managers own problems facilitate both engagement and impact (Wagner *et al*., 2017). Feedback messages should focus development opportunities related to tasks and outcomes, rather than communicating value statements about providers (Shute, 2008). This is in sharp contrast to a summative approach, in which measures and feedback messages are seen as final answers.

Social interactions between managers and professionals facilitate a formative use of measures. Measures can then be combined with qualitative and experience-based information. To find time and involve professional´s in discussion of feedback messages and its implications will be a major challenge for managers. Lack of time have been identified as an important barrier for innovative work in health care services in general (Greenhalgh *et al*., 2004). Performance management to support innovative changes will be in constant competition with pressures focusing productivity within the present clinical system.

### 5.3 The source and modality of feedback messages

Social-approval mechanisms clarify the importance of a legitimate and trusted source of feedback messages, i.e. to get feedback from someone that employees admire and identify as a role model (Ellingsen and Johanesson, 2008). In health care, collegial forms of feedback from senior professionals that fully understand the work and its contingencies is usually preferred at the clinical level. The importance of feedback from a trusted source is also identified as important in systematic reviews of audit and feedback interventions. The same principles can potentially be used when providing feedback that supports innovative change in health care services. Senior professionals with the relevant experience will not necessarily have access to extrinsic rewards and sanctions, as would managers representing the administrative hierarchy. If the theory behind behavioral change is an appeal to professional identities and social determinants of motivation, this limitation will be less important. A possible drawback is that it can be more problematic to develop a trustful relation with managers if feedback comes from senior health professionals only. A trustful relationship is more likely to develop if professionals understand actions by managers, which in turn requires continuous interaction and dialog. A team approach when providing feedback, or senior professionals acting on behalf of managers, are alternative options.

A group approach to facilitate social interactions may also be preferred on the recipient side. Studies suggests that feedback conducted with several recipients in group-settings facilitate capabilities to interpret data and identify effective action opportunities (Cooke *et al*., 2018). When receiving feedback messages in isolation, health professionals often fail to understand data and are unable to identify actions to improve quality (Desveaux *et al*., 2021). In this context it can be noted that interactive workshops together with feedback from peers and experts have been referred to as one of the most effective interventions to develop leadership in healthcare (Geerts *et al*., 2020). How to organize social interactions across managers, to facilitate understanding of feedback messages and motivation to change, is an important governance and leadership challenge.

### 5.4 The degree of transparency in feedback messages and comparisons with others

Transparency can potentially increase the impact of feedback messages. Feedback without transparency rely on self-approval mechanisms. With transparency, i.e. if other colleagues, external stakeholders and possibly the society-at-large have access to data and comparisons, social-approval mechanisms are added. More generally, empirical studies of transparent comparison of quality across health care providers confirm that professionals care about their reputation even if not linked to extrinsic incentives (Kolstad, 2013; Bevan *et al*., 2019). As presented in section 5.3, studies suggest that feedback provided in groups and allowing social interactions is associated with more effective action. In part this can be explained by reference to social-approval mechanism. Important and largely unanswered questions are when transparency is good or bad and how “it depends”. A higher level of transparency can result in significant levels of unpleasantness and defensive actions, but this can be accepted for tasks that should be well learned (Ellingsen and Johanesson, 2007). For novel tasks – including innovative change in the delivery of services–there is usually a large gap between actual and ideal performance. This suggest that transparency should be handled with care with a focus on learning rather than accountability. A related question is if transparency should include external stakeholders, or even the society at large. Based on the ideas of social-approval mechanisms and the importance of professional identities, transparency within the professional community and across managers with the same responsibility may be good enough. From one perspective, there is reasons to accept variation in transparency depending on recipient awareness and to avoid fear and worsening of self-efficacy (Landis-Lewis *et al*., 2015). Such policies will however be more demanding from an administrative perspective; providers exposed to more transparency than others may consider it unfair.

## 6. Discussion

The new approach outlined in Section 5 is not intended to replace clinical auditing and feedback interventions or monitoring of provider´s compliance towards contracts with payers. The outlined approach do clarify, however, that performance management systems focusing compliance toward clinical targets and/or contractual obligations are unlikely to support innovative changes in health care services. A key question is why health care providers would initiate change when exposed to regular feedback messages. When performance measures and targets are linked to financial incentives the answer is that otherwise they will lose out economically. In the absence of financial incentives, providers are instead assumed to be motivated intrinsically and/or through self- and social-approval mechanisms. As presented in Section 5, it is possible to design a performance management system that specifically support innovative changes through these determinants of motivation. The operationalization of an enabling approach to performance management address key components and provide a narrative of important mechanisms.

To a very high degree, the new approach outlined is an appeal to professionalism and the devotion to doing good work rather than economic reward (Freidson, 2001). Similar to other forms of control, professionalism will be an imperfect form of governance (Fournier, 1999). Bevan and Hood (2006) distinguish between four categories of health professionals: saints, honest triers, reactive gamers and rational maniacs. These categories suggest how the motivation to perform and change will vary depending on the orientation of individuals, even if tasks are interesting and important for society. Leaving the rare occasions of rational maniacs aside, these categories can be seen as an extension of Le Grands distinction between knights and knaves (Le Grand, 2003). Saints (as well as Knights) are competent and have a strong public service ethos and voluntary driving force. For these providers, feedback messages itself create a motivation to learn and change. Possibilities to determine goals and actions plans facilitates a locus of control in line with support of autonomy and intrinsic motivation (Ryan and Deci, 2000). Honest triers are less capable and need more support but are at least not inclined to manipulate data or their practice to report good performance. Reactive gamers will on the other hand look for every opportunity to game the system. This category would be difficult to handle when performance management have an enabling approach and when accountability is processual in nature. Reactive gamers may say that they are committed to a formative use of measures and changes in the delivery of services based on their own goals and actions plans, but act with far less ambition. Reactive gamers can on the other hand be even more problematic when using financial incentives within a coercive control framework. Providers can then easily play the game of “reaching the target but missing (or not caring about) the point”.

Since it cannot be expected that all providers will behave as saints or honest triers, a readiness to use some form of coercive control probably have to exist. This has been referred to as a reciprocal form of governance (Bevan *et al*., 2019). The possibility to enforce action plans and use sanctions signals an important message to providers in general, even if never used in practice. For a majority of professionals’, a reciprocal policy may be welcomed and seen as something that contributes to fairness and counteracts free-riding by reactive gamers that threatens self-governance in general. However, as has been described by Falk and Kosfeld (2006), “trusting a bit is likely to be interpreted [by employees] as not trusting at all” (p. 1629). Externally imposed forms of coercive control may also crowd out existing social norms and collective actions that enforce sanctions for free-riders by social disgrace (Ostrom, 2000).

How to combine an enabling approach with even small elements of coercive control when supporting innovative changes is an important managerial and leadership challenge. A combination of coercive and enabling control can be found in many if not all organizations (Ahrens and Chapman, 2004) and a balanced use can create dynamic tensions that contribute to organizational capability and change (Mundy, 2010; Bedford, 2015). When organizational members have a strong professional orientation, the risk of a “clash of cultures” increase (Abernethy and Stoelwinder, 1995). Bureaucratic and professional controls have since long been identified as problematic to combine (Ouchi, 1979). As most individuals have a strong preference for fairness, professionals may accept externally imposed elements of coercive control to reach that end. A possible option is to co-develop elements of coercive control together with professionals, as this may allow social norms and control of free-riders to evolve collectively, reducing the risk of crowding out (Ostrom, 2000). A key question is how professionals perceive intentions behind managerial interventions. Reciprocity should be expected if professionals perceive intentions as exploitative or in conflict with professional norms. Having “good intentions” may indeed be more important than a perfectly designed performance management system. If professionals do not perceive that intentions are “good”, an appeal to social determinants of motivation and the professional identity is not likely to work. It is interesting to note that findings from the related field of empirical studies of external inspections in health care services (used for accreditation, certification and regulation purposes) suggest that the way inspections are conducted and perceived by recipients are important for effects (Hovlid *et al*., 2020). Inspections can contribute to social interaction and reflection that improve recipients understanding of the clinical system, but only to the extent that reports are perceived as valid and reliable and conducted by a team with knowledge and communication skills that increase confidence in the process.

An additional concern is that contextual factors at the organizational and/or system level can create barriers for innovative changes in the delivery of services, even if motivation across professionals and their operational managers exist. Saints and honest triers may even feel forced to develop into reactive gamers due to organizational shortages. As pointed out by Malmmose and Kure (2020) a new role for managers using an integrated set of performance measures demand changes at the institutional level. Professionals and their managers need leadership support and the opportunity to change. Financial incentives can act as barriers to change even if nonfinancial incentives are used within the context of performance management. To allow innovative changes in the delivery of services, payment systems and resource allocation need to be flexible enough, e.g. using different forms of bundled, capitated or comprehensive payments (see, e.g. Ryan, 2018; National Academies of Sciences Engineering and Medicine, 2021). Bundled, capitated and comprehensive payment systems can however only provide opportunity for innovative changes in the delivery of services. The motivation to change will need to rely on leadership and management that fully recognize determinants of motivation and its implications for performance management.

## 7. Conclusion

This article suggests how an enabling approach to performance management can support innovative changes in the delivery of services. Such complex changes can rarely rely on a centralized linear approach with implementation of given standards. Key design elements explored are support of local goals and action plans that fit specific conditions and challenges, a formative and learning oriented use of measures and an appeal to professional identities and self- and social-approval mechanisms when providing feedback. The approach is consistent with new governance and managerial approaches emerging in public sector organizations more generally, supporting a higher degree of professional autonomy and use of nonfinancial incentives. Several management and leadership challenges as well as research opportunities exist. Similar to other forms of control, an appeal to professionalism is an imperfect form of governance. A continued debate about the interpretation of performance measures and performance management in health care services can be expected.
